# Mesenchymal Stem Cells for the Compassionate Treatment of Severe Acute Respiratory Distress Syndrome Due to COVID 19

**DOI:** 10.14336/AD.2020.1218

**Published:** 2021-04-01

**Authors:** Martin Iglesias, Patricia Butrón, Iván Torre-Villalvazo, Erik A Torre-Anaya, Juan Sierra-Madero, José J Rodriguez-Andoney, Armando R Tovar-Palacio, Alejandro Zentella-Dehesa, Guillermo Domínguez-Cherit, Tatiana S Rodriguez-Reyna, Julio Granados-Arriola, Verónica Espisosa-Cruz, Fernando P Téllez-Pallares, Alexia Lozada-Estrada, Carol A Zepeda Carrillo, Aldo J Vázquez-Mézquita, Hector F Nario-Chaidez

**Affiliations:** ^1^Plastic and Reconstructive Surgery Service at Instituto Nacional de Ciencias Médicas y Nutrición Salvador Zubirán, México.; ^2^Nutrition Physiology Department at Instituto Nacional de Ciencias Médicas y Nutrición Salvador Zubirán, México.; ^3^Infectology Department at Instituto Nacional de Ciencias Médicas y Nutrición Salvador Zubirán, México.; ^4^Intensive Care Unit Department at Instituto Nacional de Ciencias Médicas y Nutrición Salvador Zubirán, México.; ^5^Biochemistry Laboratory at Instituto Nacional de Ciencias Médicas y Nutrición Salvador Zubirán, México.; ^6^Rheumatology Department at Instituto Nacional de Ciencias Médicas y Nutrición Salvador Zubirán, México.; ^7^Inmunogenetic and Transplant Department at Instituto Nacional de Ciencias Médicas y Nutrición Salvador Zubirán, México.; ^8^Radiology and Imaging Department at Instituto Nacional de Ciencias Médicas y Nutrición Salvador Zubirán, México.; ^9^Tecnológico de Monterrey, Escuela de Medicina y Ciencias de la Salud, México.; ^10^Fellow-Clerk of Plastic Surgery at Instituto Nacional de Ciencias Médicas y Nutrición Salvador Zubirán, México.; ^11^Radiology and Molecular Imaging Department at the American British Cowdray Medical Center.; ^12^Head of Mesenchymal Stem cell Therapy Department of CBCells Biotechnology.

**Keywords:** mesenchymal stem cell, stromal cell, COVID-19, ARDS, cell transplantation

## Abstract

Mesenchymal stem cells (MSC) have received particular attention due to their ability to inhibit inflammation caused by cytokine storm induced by COVID-19. In this way some patients have been treated successfully. The aim of this study was to evaluate the safety and describe the clinical changes after IV administration of allogeneic human umbilical cord MSC (ahUCMSC), in patients with bilateral pneumonia caused by COVID-19, complicated with severe ARDS, as compassionate treatment. This was a pilot, open-label, prospective, longitudinal study. Five patients that did not improve in their clinical conditions after 48 hours of receiving the standard medical management used by the Medical Center and with persistent PaO2/FiO2 less than 100 mmHg were enrolled. ahUCMSC were infused IV, at dose of 1x10^6^ per Kg of body weight over 15 minutes. Patients were monitored after the infusion to detect adverse event. Pa02/FiO2, vital signs, D-dimer, C reactive protein and total lymphocytes were monitored for 21 days after the infusion or until the patient was discharged from the hospital. Descriptive statistics were used with means or medians and standard deviation or interquartile range according to the type of variable. The Wilcoxon’s rank-sum was used for stationary samples. Adverse events occurred in three patients and were easily and quickly controlled. Immediately after the infusion of ahUCMSC, constant rise of PaO2/FiO2 was observed in all patients during the first 7 days, with statistical significance. Three patients survived and were extubated on the ninth day post-infusion. Two patients died at 13 and 15 days after infusion. The infusion of ahUCMSC in patients with severe ARDS caused by COVID-19, was safe, and demonstrated its anti-inflammatory capacity in the lungs, by improving the respiratory function expressed by PaO2 / FiO2, which allowed the survival of 3 patients, with extubation at 9 days.

The COVID-19 fatality rate ranges from 0.00% to 1.63% (www.who.int/bulletin/online_first/BLT.20.265892.pdf). Approximately 6% to 15% of patients will progress to critically severe acute respiratory distress syndrome (ARDS), which is the leading cause of death and which requires care in the intensive care unit (ICU). Of these critically ill patients, 2.5% will die [1,2]. These patients who die mainly comprise elderly patients, particularly those aged >80 years (15%) and 70 to 79 years (8%). Death also occurs in approximately half of critically ill patients with pre-existing comorbidities such as cardiovascular disease, diabetes, chronic respiratory disease, and oncological disease [2,3]. The survival time after ICU admission is generally 2 weeks [4]. As of March 17, 2020, in one study, mortality in a group of 21 patients with ARDS due to COVID-19 was 67%, and 24% of the patients remained critically ill; only 9.5% had been discharged from the ICU [3].

Covid-19 is a unique disease characterized by a strong innate immune response that requires effective immune regulation. This can be reached in 80% of infected individuals. However, in the remaining 20%, the SARS-CoV-2 virus induces a severe form of the disease which is characterized by an overactive immune response that produces a cytokine storm in the lungs, mononuclear cell activation, lymphopenia (both CD4+ and CD8+ subsets) and unregulated inflammation that leads to thrombotic microangiopathy involving unregulated complement activation, within the first 4-6 days after illness onset [1,2, 5-7]. This cytokine storm induces pulmonary edema, air exchange dysfunction, ARDS, acute cardiac injury, multi-organ failure and generally, secondary infection leading to death [1]. However, much of the pathophysiology of SARS-CoV2 is still undefined [8].

There is currently no specific cure for COVID-19. Thus, the clinical management of these patients involves supportive care, supplemental oxygen, infection prevention or control, and mechanical ventilation, when necessary [8]. Novel strategies to increase the pulmonary protective efficacy of antensin-converting enzyme 2 (ACE-2)/Mas-receptor pathway are being implemented using administration of drugs that directly or indirectly raise or regulate the levels of ACE-2. Some drugs that are being explore are aldosterone, hidro-chlorothiazide, vasaltan, resveratrol, liraglutide, linagliptin and other unproved drugs as xanthenone, resorcinol naphthalene etc. [9]. Non-specific antivirals, antibiotics to treat secondary infections and sepsis, steroids to reduce inflammation, immunoglobulins, intravenous sera transfusion from recovered patients, and cytokine receptor blockers to treat severe ARDS caused by COVID-19 have not provided consistent positive results [1,4,10]. Liu et al. reported 109 COVID-19 infected patients; 48.6% of the patients developed ARDS, and no significant effect on survival was observed despite the use of antivirals, glucocorticoids, or immunoglobulins [11]. Therefore, multiple investigations of various drugs for the treatment of COVID-19 are on-going [2,5,12].

Mesenchymal stem cells (MSC) have received particular attention due to their ability to inhibit inflammation caused by cytokine storm and because of their immunoregulatory capacity, which have been shown in several in vitro and in vivo models [13,14]. Clinically, the safety of MSCs as well as their immunoregulatory properties have been demonstrated in multiple pathologies, such as cardiovascular disease, inflammatory bowel disease, diabetes mellitus, kidney diseases, osteoarthritis, bone regeneration, cirrhosis, multiple sclerosis, systemic lupus erythematosus, graft-versus-host disease, and ARDS [2,4,15]. MSCs have immunoregulatory properties, increasing regulatory cluster of differentiation CD4 and CD8 T cells and M2 macrophages, and blocking the presentation of antigens (SARS-CoV2) by mature dendritic cells. Additionally, MSCs have antiapoptotic properties in injured cells, aid in clearing alveolar exudate, and can restore the alveolar endothelium and epithelium, all of which decrease the probability of pulmonary fibrosis and preserve long-term lung function. MSCs also have antibacterial, antiviral, and analgesic properties [4,11]. These immunomodulatory and anti-inflammatory properties of MSCs were confirmed in the treatment of respiratory diseases in 17 completed clinical studies [8]. For this reason, MSCs have become a therapeutic approach in treating ARDS due to COVID-19, and many clinical trials have been registered [2,5,8,16,17] (https://clinicaltrials.gov).

MSCs can be isolated from various tissues such as the bone marrow, adipose tissue, umbilical cord, dental pulp, menstrual blood, buccal fat pad, and fetal liver. All of these tissues have the same above-mentioned properties [8].

Umbilical cord MSC (UCMSC) have the following properties: a high concentration of cells, fast doubling times in the laboratory, noninvasive extraction, and a gene expression profile similar to that of embryonic stem cells (which means they have fast doubling times, high plasticity, and possibly high potency). Thus, UCMSC seem to be optimal for the treatment of coronavirus [3].

Allogeneic human UCMSC (ahUCMSC) are immune-evasive. They express low levels of major histocompatibility complex class I but not class II molecules on their cell surface, allowing their transplantation [3].

However, despite the research findings, MSC therapy in COVID-19 has been reported in few patients. Leng et al., reported seven patients with clinical COVID-19 pneumonia who received a single intravenous dose of clinical-grade adipose MSCs at a dose of 1 × 10^6^ cells per kilogram of body weight [18]. Liang et al., reported a 65-year-old woman with COVID-19 pneumonia, in critical condition, with tracheal intubation and liver damage. She was treated with ahUCMSC as three intravenous infusions of 5 × 10^6^ahUCMSCs/Kg of body weight every 3 days [19]. The patients in both studies survived, no adverse events were reported, respiratory symptoms improved in 2-4 days, and inflammatory cytokine concentrations decreased significantly.

On April 24, 2020, the Australian company, Mesoblast, reported the treatment of 12 patients with COVID-19 who were ventilator-dependent with moderate to severe ARDS. Patients were treated with two administrations of stem cells derived from bone marrow, but the report did not include the dose. The survival rate was 83%, and nine patients were successfully extubated 10 days post-infusion (www.bioworld.com/articles/434640-mesoblast-reports-83-survival-in-ventilator-dependent-covid-19-patients-following-stem-cell-therapy).

Pluristem Therapeutics Inc. (MSCs derived from placenta) reported the treatment of 7 patients with ARDS who required ventilator support; 100% 7-day survival was reported (www.globenewswire.com/news-release/2020/04/07/2012754/0/en/Pluristem-Reports-Preliminary-Data-from-its-COVID-19-Compassionate-Use-Program-Treating-Seven-Patients-with-Acute-Respiratory-Failure.html).

Due to the qualities of MSCs already mentioned, there are currently 37 clinical trials registered at ClinicalTrials.gov in which MSCs from different sources will be used to treat different COVID-19 stages. https://clinicaltrials.gov For this reason, we designed this study to evaluate the safety and describe the clinical changes after intravenous (IV) administration of ahUCMSC in patients with bilateral pneumonia caused by COVID-19, complicated with severe ARDS, as compassionate treatment.

## MATERIAL AND METHODS

This was a pilot, open-label, prospective, longitudinal study. The protocol was approved by the Research Committee and by the Research Ethics Committee of our institution (approval code: SCI-3354-20-21-1), and the study was registered at ClinicalTrials.gov with the code NCT04416139. Five patients of either sex, older than 18 years of age, with bilateral pneumonia caused by COVID-19, complicated with severe ARDS according to the Berlin definition [20], with positive SARS-CoV2 polymerase chain reaction (PCR) ribonucleic acid (RNA), and with no clinical improvement after 48 hours of receiving the standard medical management used by the medical center were enrolled. Patients met the following additional inclusion criteria: a) persistent partial pressure of arterial oxygen/fraction of inspired oxygen (PaO2/FiO2) < 100 mmHg; b) persistent fever; c) an increase in D-dimer concentrations by at least 50% from the baseline value and/or ferritin concentrations > 1000 ng/mL; d) chest computed tomography imaging showed ground-glass opacity and bilateral pneumonia; and e) sequential organ failure assessment (SOFA) score < 11. During this study, patients received the standard management measures used at that time by the medical institution. The exclusion criteria were a) pneumonia caused by COVID-19 complicated with mild and moderate ARDS, and b) moribund patients not expected to survive the next 48 hours. ahUCMSC was administered after informing family members that this was a compassionate procedure, and after obtaining informed signed consent.

Clinical grade ahUCMSC was donated by CBCells BioTechnology, with registration and approval of the Federal Commission for the Protection against Sanitary Risks, license number 18-TR-14-120-0001, with the product processed according to good manufacturing practice. (GMP) The panel of cell surface markers is shown in a Supplementary Table 1. ahUCMSC were infused IV, at a dose of 1 × 10^6^ per Kg of body weight over 15 minutes. Patients were monitored for 4 hours after ahUCMSC infusion to detect prespecified infusion-associated events and/or any other unexpected adverse event [15].

Pa02/FiO2, body temperature, respiratory rate (RR), and heart rate (HR) were monitored daily for 21 days after the infusion or until the patient was discharged from the hospital. Total lymphocyte counts, and serum D-dimer and C-reactive protein (CRP) concentrations were evaluated every 3 days.

Non-contrasted chest CT scans were performed before and after infusion to evaluate the progression of patient’s pneumonia. Semi-automatic lung segmentation and volumetry of the CT scans were performed with the software 3D Slicer, Version 4.11. Normal or undamaged parenchyma is defined as the “portion of the lung exclusive of visible pulmonary vessels and airways”. A ground-glass opacity is defined as “hazy increased opacity of lung, with preservation of bronchial and vascular margins”. While consolidation is defined as a “homogeneous increase in pulmonary parenchymal attenuation that obscures the margins of vessels and airway wall”. These definitions were obtained from the “Glossary of Terms of Thoracic Imaging” by the Fleischner Society [21]. Ground-glass opacities and consolidations were considered as damaged parenchyma.

**Figure 1.**
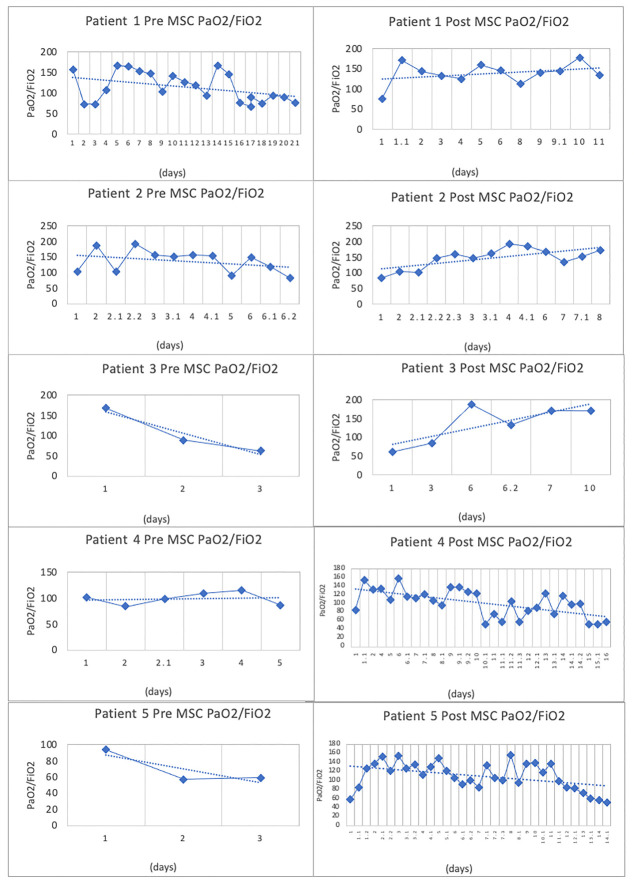
The total respiratory evolution of the patients according to the values of PaO2/FiO2.

**Table 1:** Demographic Data and Medical Status

Patient data	Patient 1	Patient 2	Patient 3	Patient 4	Patient 5
Age (years)	34	58	66	62	43
Sex	male	female	male	male	male
Weight (Kg)	116	78	60	80	117
Height (cm)	162	149	165	170	170
BMI	44.2	35.3	22	27.6	40.4
Comorbidity	Morbid Obesity	DM II, AH, Morbid Obesity, PAD, hT, Dyslipidemia	DM II, Pulmonary Fibrosis	Overweight	Morbid obesity
SOFA	6	7	4	6	6
PaO2/FiO2 Pre infusion	76^*^	84^*^	62^**^	86^*^	59^*^
ARDS	Critically Severe	Critically Severe	Critically Severe	Critically Severe	Critically Severe
PCR SARS COV2	(+)	(+)	(+)	(+)	(+)

* Invasive mechanical ventilation.

** BIPAP 15 l x min. AH: Arterial Hypertension. PAD: Peripheral artery disease. hT: Hypotiroidism. DM II: Diabetes mellitus Type II

### Statistical analysis

Numerical variables are reported as mean ± standard deviation or median with interquartile range. Categorical variables are reported as percentages. The Kruskal-Wallis test was performed to compare the effect of MSCs on the PaO_2_/FiO_2_ ratio at the different assessment points. A two-tailed P value of <0.05 was considered statistically significant. Analyses were performed with STATA statistical software, version 14.1 (StataCorp, College Station, TX, USA).

**Figure 2.**
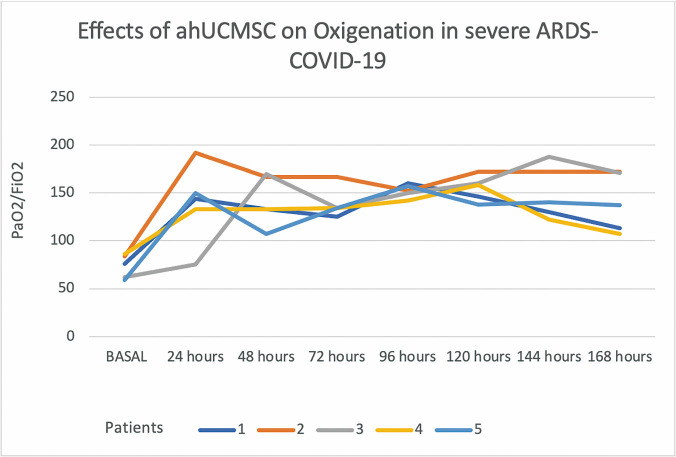
The respiratory evolution of the patients during the first seven days after the infusion of ahUCMSC according PaO2/FiO2.

**Figure 3.**
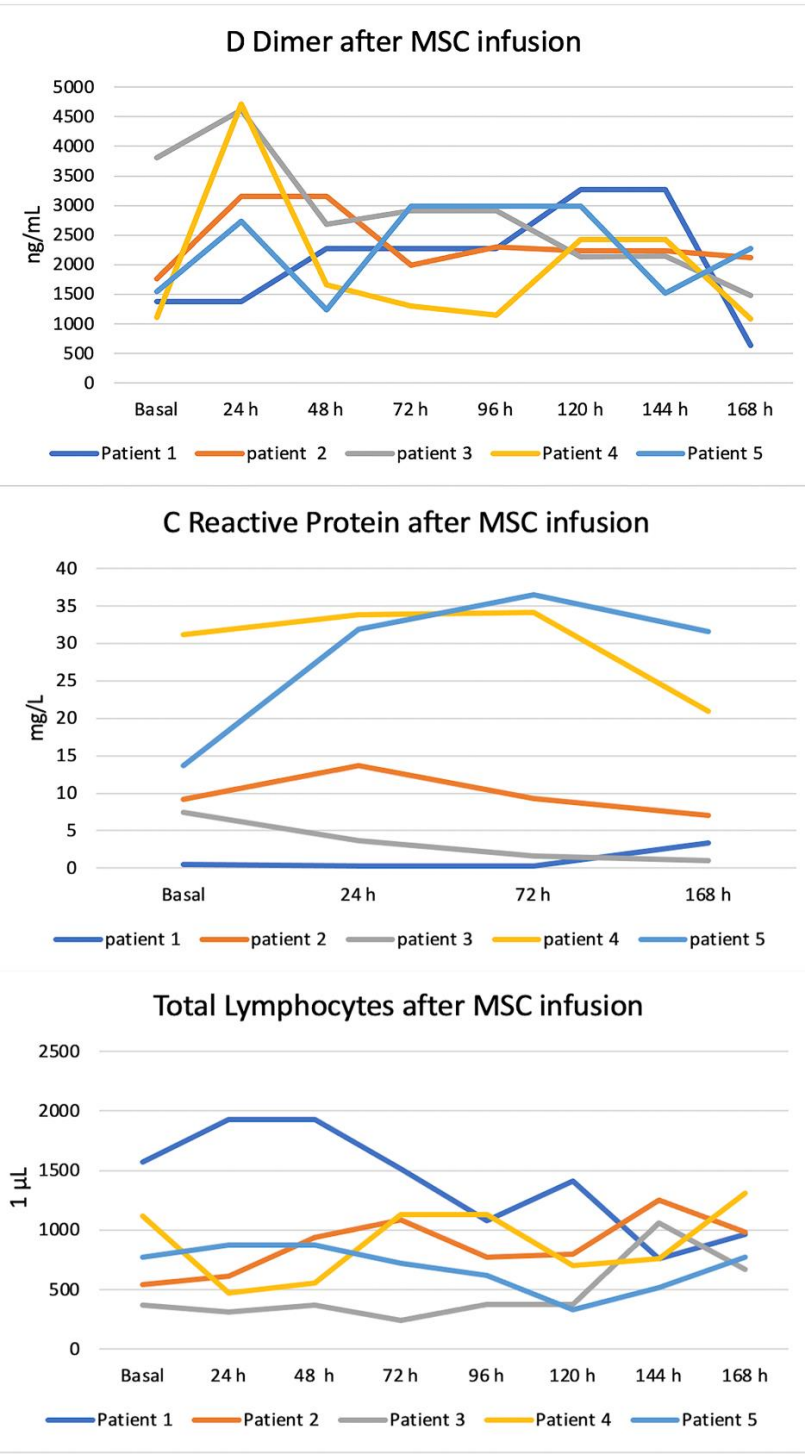
Biochemical evolution of the patients after ahUCMSC infusion.

## RESULTS

Patients’ demographic data and medical status are presented in Table 1. All patients were critically and severely affected with ARDS, with comorbidities. Four of the patients required ICU care and were ventilator dependent. Patient 3 had the absolute indication for hospitalization in ICU, however at that time there were no beds available in ICU and he was treated in regular hospitalization with the consequences that this may have caused. Prespecified infusion-associated events developed during the first hour post-infusion in patients 4 and 5. These patients developed hypoxemia and hypotension and/or hypertension; patients 3 and 4 developed muscle spasms. All adverse events were easily and quickly controlled and did not acutely alter patients’ medical conditions. Progressive disease findings and tracheal intubation time in the days before ahUCMSC infusion, adverse events after ahUCMSC infusion and the duration and treatment for the adverse events, total ICU stay and ICU stay after the ahUCMSC infusion, complications due to COVID-19, and the final results, are presented in Table 2.

**Table 2:** Infusion, Adverse Events and Final Outcomes.

	Patient 1	Patient 2	Patient 3	Patient 4	Patient 5
Days pre infusion disease/Intubated	27(17)	12(6)	22	10(4)	16(1)
Adverse Events	none	none	Muscle contractions in extremities	Muscle contractions in extremities and chest, PO2 decreased 78%, AH^1^, respiratory effort	Hypotension
Treatment	none	none	none	Propofol, Increasing FiO2	vasopresin
Duration (min)	0	0	15	5	60
Total days in ICU/after MSC^*^	27(10)	18(12)	0	19(15)	14(13)
Complications due to COVID	Paralytic Ileus Mixed Delirium	Bacterial Pneumonia Cardiomyopathy	Hypoactive Delirium	Cardiomyopathy, Liver Failure Bacterial Pneumonia Active bleeding, LLEAT^2^	AKI^3^ KDIGO III Active bleeding
Status	Alive	Alive	Alive	Dead	Dead

* MSC: Mesenchymal stem cells.

1 AH: Arterial Hypertension.

2 LLEAT: Left Lower Extremity Arterial Thrombosis.

3 AKI: Acute Kidney Injury

**Table 3:** Oxigenation after ahUMSC ^*^ infusion.

Time after ahUCMSC infusion (hours)	PaO2/FiO2 Median (IQR)	*P* value (0.05)^**^
Basal	76 (62-84)	-
24	144 (133-150)	0.047
48	142 (133-167)	0.008
72	138 (133-134)	0.008
96	152 (150-157)	0.009
120	154 (146-160)	0.009
144	154 (140-172)	0.009
168	140 (113-171)	0.019

* ahUCMSC= Allogeneic human umbilical cord mesenchymal stem cell

** P= 0.05, The Kruskal-Wallis Test

Three patients survived, and two patients died. Hemoglobin concentration decreased to 7.2 g/dl 14 days post-infusion in patient 4. Anticoagulation therapy was suspended, and two units of red blood cells were transfused. Fifteen days post-infusion, patient 4 developed arterial thrombosis in his left lower limb, hemodynamic deterioration, D-dimer concentration of 7268 ng/mL, and death. *Enterobacter cloacae* was cultured in endotracheal aspiration samples. After admission to hospital, patient 5 developed progressively increased serum creatinine concentrations of 2.51, 3.71, 5.02, and 7.14 mg/dL, for which hemodialysis was performed incompletely due to hemodynamic alterations. Eleven days post-infusion, this patient developed epistaxis and hematuria, and anticoagulation therapy was suspended. The patient died 13 days post-infusion.

PaO2/FiO2 values decreased in all patients during the pre-infusion stage. Figure 1 shows that immediately after infusing ahUCMSC, PaO2/FiO2 values increased progressively and significantly over the following 7 days (Table 3 and Fig. 2).

All patients developed increased D-dimer concentrations after the first 24 hours post-infusion of between 2738 ng/mL and 4712 ng/mL. After this time, D-dimer concentrations decreased; however, there were value fluctuations due to patients’ added complications. CRP concentrations remained normal in patients 1, 2, and 3 during the first 7 days. In the patients who died, CRP concentrations increased during the same period. Total lymphocytes were minimally elevated 7 days post-infusion. Only patient 1 had a decrease in total lymphocytes from 1570 to 984 per 1 ML. (Fig. 3). The pre- and post-infusion evolution of the clinical cases has been included as Supplementary Fig. 1 and Supplementary Clinical Cases.

**Figure 4.**
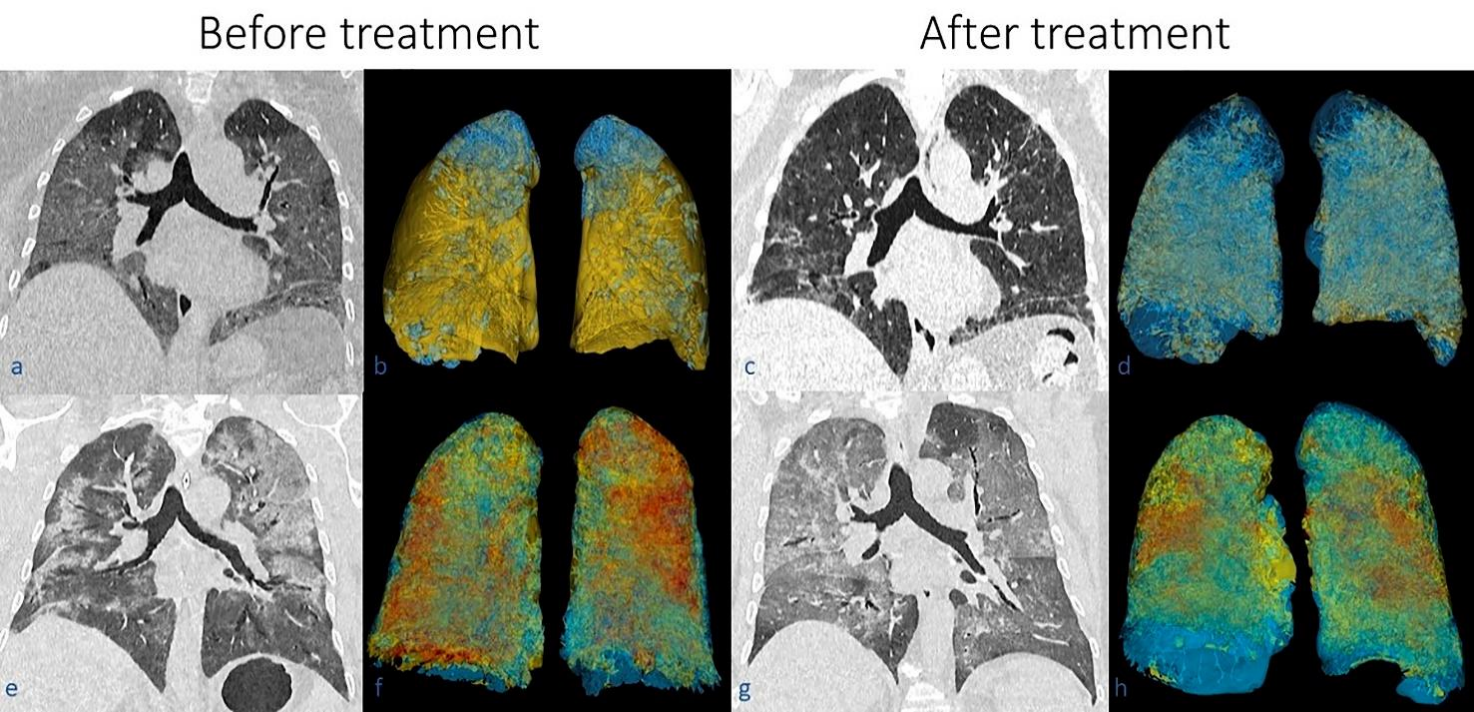
The images of the first row correspond to patient 1 (a, b, c, d), while images of the second row correspond to patient 4 (e, f, g, and h). CT scans in coronal plane are shown before (a, e) and after treatment (c, g) with their respective three-dimensional segmentation (b, and f before treatment; c and g after treatment). Notice in the affected lung parenchyma in the CT scans as ground-glass opacities and consolidations. In the three-dimensional lung segmentation, blue corresponds to undamaged parenchyma, the yellow color corresponds to ground-glass opacities, while the red color corresponds to consolidation. Notice the reduction of ground-glass opacities and consolidations in the lungs of both patients after treatment.

The chest CT scan volumetry results that consider the percentage of damaged parenchyma before and after infusion are listed in Table 4. A three-dimensional representation of the damaged and undamaged lung parenchyma of two representative cases is presented in Figure 4.

## DISCUSSION

Due to their anti-inflammatory and immunomodulatory properties, MSCs, whether autogenous or allogeneic and with different origins, have been used prior to the COVID-19 pandemic to treat multiple severe respiratory diseases. Previous studies have reported the absence of adverse events and improved respiratory function with long-term pulmonary function preservation after MSC therapy [4,15]. Wilson et al reported using MSCs to treat non-COVID-19 ARDS and also reported improved respiratory function without adverse events, but without major changes in inflammatory markers or mortality, when the studies were controlled [15]. These findings are why allogeneic MSCs administered IV are an emerging treatment in COVID-19.

**Table 4:** Total volume pulmonar undamage and damage by Covid-19.

Patient	Infusion Stage	Total lung volume (cc)	Undamaged lung parenchyma (cc/%)	Damaged lung parenchyma (cc/%)
1	Pre	1914	202 (10.6)	1712 (89.4)
	Post^1^	2262	1517 (67.1)	745 (32.9)
2	Pre	1777	1280 (72.0)	497 (28.0)
	Post^1^	2462	2182 (88.6)	280 (11.4)
3	Pre	3398	2059 (60.6)	1339 (39.4)
	Post^2^	2550	1846 (72.4)	704 (27.6)
4	Pre	3914	1582 (40.4)	2332 (59.6)
	Post^3^	3239	1019 (31.5)	2220 (68.5)
^*^5	Pre	2016	371 (18.4)	1645 (81.6)

1. Three-weeks post infusion;

2. Two weeks post infusion;

3. One-week post infusion

*. Patient died before the follow-up CT scan.

Currently, there are only preliminary reports showing the possible usefulness of MSCs in the treatment of patients with pneumonia due to COVID-19 complicated with ARDS, and the main objective of these reports was to evaluate the safety of administering allogeneic MSCs before proceeding to large controlled clinical studies [18,19] (www.bioworld.com/articles/434640-mesoblast-reports-83-survival-in-ventilator-dependent-covid-19-patients-following-stem-cell-therapy; www.globenewswire.com/news-release/2020/04/07/2012754/0/en/Pluristem-Reports-Preliminary-Data-from-its-COVID-19-Compassionate-Use-Program-Treating-Seven-Patients-with-Acute-Respiratory-Failure.html).

These studies reported an absence of adverse events and significant improvement in respiratory symptoms and inflammatory markers 2-4 days post-infusion [18,19]. However, in Leng et al’s report, none of the treated patients presented with ARDS. Likewise, ARDS in the patient in Liang et al’s study was not classified according to the Berlin classification. Mesoblast used MSCs in moderate to severe ARDS patients, but without specifying the number of patients in each group. The Pluristem company also did not define the severity of ARDS well, in their study.

As in previous studies, our main objective was to evaluate the safety of ahUCMSC administration, as a compassionate treatment, in patients with bilateral pneumonia caused by COVID-19 who were complicated by severe ARDS according to the Berlin classification, using PaO2/FiO2 as an indicator. Although the ahUCMSCs met the GMP criteria, and all of our patients received anticoagulant therapy, patients 4 and 5 developed prespecified infusion-associated events as reported by Wilson [15]. These patients required an increase in FiO2 and vasopressor treatment. Patients 3 and 4 developed muscle spasms, and one required treatment with a sedative. Fortunately, all of these adverse events were easily controlled. The presence of adverse events can be explained by the severity of the patients’ conditions; therefore, severely-affected patients should be monitored closely for 4-6 hours after ahUCMSC infusion. We consider that the ease of control and short duration of the adverse events indicates the safe application of ahUCMSC in these patients.

The beneficial effect of IV infusion of allogeneic MSCs on respiratory function has been reported to be almost immediate. Liang *et al* reported improved respiratory symptoms 2-4 days post-infusion [18,19]. Pluristem Therapeutics Inc. reported improvement in respiratory function after 7 days of follow-up, with 100% survival. Likewise, our five treated patients showed constant and progressive improvement in PaO2/FiO2 from 24 hours after the ahUCMSC infusion until the 7th day, demonstrating an immediate beneficial action of MSCs on respiratory function.

The patient reported by Liang et al. was extubated 4 days after the first infusion [19]. Mesoblast reported extubation in 9 of 12 patients on the 10th day post-infusion, and 7 were discharged from the hospital at this time; the authors reported 83% survival. Pluristem Therapeutics Inc. reported that of seven patients, three were being extubated on the 7th day post-infusion. Two of our patients were removed from ventilator support 9 days post-infusion. Therefore, we consider that if a patient with severe ARDS responds positively to an MSC infusion, and does not develop additional complications (e.g., multi-organ failure), the patient will be extubated between 4- and 10-days post-infusion.

The majority of the author have reported improvements in CRP, D Dimer, HB, Lymphocytes, and inflammatory markers [18,19,22]. Only Barkama et al, have reported improvements in PaO2/FiO2 [18]. In our study CPR, D Dimer, Lymphocytes showed values depending on the presence of complications caused by COVID. Perlee et al., noted transient increases in the concentrations of plasma thrombin-antithrombin complexes and D-dimers, after allogeneic adipose-derived MSC [23]. This event was also observed in our study after ahUCMSC infusion.

Leng et al., reported a decreased concentration of the serum proinflammatory cytokine tumor necrosis factor-α and increased concentrations of interleukin 10 and vascular endothelial growth factor after infusion of MSC [18]. Liang et al., reported that the CD3+ T cell, CD4+ T cell, and CD8+ T cell counts also markedly increased to normal levels [19]. These data support the anti-inflammatory and immunomodulatory properties of MSCs. However, these markers were not evaluated in our study, which is an important limitation. Our study focused only on the clinical changes after ahUCMSC infusion.

There is still doubt about the effective dose of MSC infusion. In non-COVID ARDS, the safe and effective dose to obtain improvement in respiratory function is 5 × 10^6^/Kg of body weight [11,15, 24]. The applied dose of MSC for treating COVID-19-related ARDS varies by study as a single infusion at a dose of 1 × 10^6^/Kg of body weight [18], three infusions (total) at a dose of 50 × 10^6^/kg of body weight every third day [19], and Mesoblast applied two MSC infusions over 5 days with no recorded dose. Our patients received a dose of 1 × 10^6^/kg of body weight as a single bolus infusion. However, despite the fact that patients’ respiratory function improved, and that three survived, we consider it prudent and necessary to increase the infusion dose. Current registered clinical studies report that they will use 3-4 MSC infusions at doses of 5 × 10^6^/Kg, while others plan doses of 0.5 × 10^6^/Kg [3]. The route of administration also differs and may be IV, intranasal, inhalational, or intratracheal.

MSC administration time is also critical. Better results have been reported with a shorter duration of ARDS or intubation. Some authors consider that MSCs should be infused within 96 h of having diagnosed ARDS [11,15]. Mesoblast infused MSCs within 72 hours after intubation, while Liang et al infused MSCs 9 days after intubation; however, blood oxygen saturation (SpO2) and PaO2/FiO2 values were not reported [19]. In our surviving patients, the infusion was performed 17 and 6 days after patients were intubated. In our patients who died, infusion was performed 3 days and 1 day, respectively, after intubation. Unfortunately, these two patients developed multi-organ failure at the onset of the disease and may have required a higher dose to control the inflammatory process.

Wilson et al and Liu et al., defined the exclusion criteria for MSC infusion in non-COVID ARDS as: pregnancy, recent deep vein thrombosis or pulmonary thromboembolism in the last 3 months, severe pre-existing organ diseases, cancer, acquired immunodeficiency syndrome (AIDS), diseases or conditions with > 50% mortality in the next 6 months, moderate or severe liver failure, major trauma within 5 days prior to the infusion, and death expectation within the next 24 hours. These criteria must be applied in ARDS caused by COVID-19 [11,15].

With the data obtained from the five indicated studies, including ours, we consider that the best indications for MSC infusion are patients with bilateral pneumonia caused by COVID-19 complicated with moderate ARDS according to the Berlin classification (PaO2/FiO2 > 100 or ≤ 200 mmHg), and we recommend infusing patients before 96 hours after a diagnosis of ARDS, or at most, 72 hours after intubation, following the exclusion criteria already indicated.

Conclusion: ahUCMSC infusion administered as compassionate treatment in patients with bilateral pneumonia caused by COVID-19 and complicated with critically severe ARDS was safe and demonstrated its anti-inflammatory capacity in the lungs by improving respiratory function expressed as PaO2/FiO2. This treatment led to the survival of three patients who were extubated 9 days post-infusion. However, the value of these data is limited due to the small sample size. We consider that MSC infusions in COVID-19 patients with moderate ARDS, in a controlled clinical study, are required to verify the usefulness of MSC infusions in these patients.

## Supplementary Materials

The Supplemenantry data can be found online at: www.aginganddisease.org/EN/10.14336/AD.2020.1218, and https://drive.google.com/drive/folders/18nsVKMRlMDphYNzgB24_mwefvvR9zeYQ?usp=sharing.



## References

[1] Metcalfe SM (2020). Mesenchymal stem cells and management of COVID-19 pneumonia. Med Drug Discov, 5:100019 32296777 10.1016/j.medidd.2020.100019 PMC7147223

[2] Rao V, Thakur S, Rao J, Arakeri G, Brennan PA, Jadhav S, et al. (2020). Mesenchymal stem cells-bridge catalyst between innate and adaptive immunity in COVID 19. Medical Hypotheses, 143: 109845. 32425307 10.1016/j.mehy.2020.109845 PMC7232064

[3] Atluri S, Manchikanti L, Hirsch JA (2020). Expanded Umbilical Cord Mesenchymal Stem Cells (UC-MSCs) as a Therapeutic Strategy In Managing Critically Ill COVID-19 Patients: The Case for Compassionate Use. Pain Physician, 23:E71-E83. 32214286

[4] Rogers C, Harman RJ, Bunnell BA, Schreiber MA, Xiang C, Wang FC, et al. (2020). Rationale for the clinical use of adipose-derived mesenchymal stem cells for COVID-19 patients. J Transl Med 18:203. 32423449 10.1186/s12967-020-02380-2 PMC7232924

[5] O’Driscoll L (2020). Extracellular vesicles from mesenchymal stem cells as a Covid-19 treatment. Drug Discov Today, 25: 1124-1125. 32387262 10.1016/j.drudis.2020.04.022 PMC7202814

[6] García LF (2020). Immune Response, Inflammation, and the Clinical Spectrum of COVID-19 Frontiers in Immunology, 11:1-13. 10.3389/fimmu.2020.01441 PMC7308593 32612615

[7] Le Bert N, Tan AT, Kunasegaran K, Tham CYL, Hafezi M, Chia A, et al. (2020). SARS-CoV-2-specific T cell immunity in cases of COVID-19 and SARS, and uninfected controls. Nature; 584: 457-462. 32668444 10.1038/s41586-020-2550-z

[8] Golchin A, Seyedjafari E, Ardeshirylajimi A (2020). Mesenchymal Stem Cell Therapy for COVID-19: Present or Future. Stem Cell Reviews and Reports,16:427-433. 32281052 10.1007/s12015-020-09973-w PMC7152513

[9] Kaur U, Acharya K, Mondal R, Singh A, Saso L, Chakrabarti S, et al. (2020). Should ACE2 be given a chance in COVID-19 therapeutics: A semi-systematic review of strategies enhancing ACE2. Eur J Pharmacol; 887: 1735-1745 10.1016/j.ejphar.2020.173545 PMC7485553 32926917

[10] Rogers TF, Zhao F, Huang D, Beutler N, Burns A, H W et al. (2020). Isolation of potent SARS-CoV-2 neutralizing antibodies and protection from disease in a small animal model. Science; 369:956-963 32540903 10.1126/science.abc7520 PMC7299280

[11] Liu S, Peng D, Qiu H, Yang K, Fu Z, Zou L, et al. (2020). Mesenchymal stem cells as a potential therapy for COVID-19. Stem Cell Research & Therapy, 11:169. 32366290 10.1186/s13287-020-01678-8 PMC7197031

[12] Richardson C, Bhagani S, Pollara G (2020). Antiviral treatment for COVID-19: the evidence supporting remdesivir. Clin Med (Lond), In press. 10.7861/clinmed.2020-0524 PMC7687338 32863273

[13] Shi Y, Su J, Roberts AI, Shou P, Rabson AB, Ren G (2012). How mesenchymal stem cells interact with tissue immune responses. Trends Immunol, 33:136-43. 22227317 10.1016/j.it.2011.11.004 PMC3412175

[14] Harrell CR, Sadikot R, Pascual J, Fellabaum C, Jankovic MG, Jovicic N, et al. (2019). Mesenchymal stem cell-based therapy of inflammatory lung diseases: current understanding and future perspectives. Stem cells Int, 2019:4236973. 31191672 10.1155/2019/4236973 PMC6525794

[15] Wilson J, Liu Kd, Zhuo H, Caballero L, McMillan M, Fang X (2015). Mesenchymal stem (stromal) cells for treatment of ARDS: a phase 1clinical trial. Lancet Respiratory Med, 3: 24-32. 10.1016/S2213-2600(14)70291-7 PMC4297579 25529339

[16] Bari E, Ferrarotti I, Saracino L, Perteghella S, Torre ML, Corsico AG (2020). Mesenchymal Stromal Cell Secretome for Severe COVID-19 Infections: Premises for the Therapeutic Use. Cells, 9: 924 10.3390/cells9040924 PMC7226831 32283815

[17] Rajarshi K, Chatterjee A, Ray S (2020). Combating COVID-19 with Mesenchymal Stem Cell therapy. Biotechnology Reports (Amst), 26:e00467. 10.1016/j.btre.2020.e00467 PMC7224671 32420049

[18] Leng Z, Zhu R, Hou W, Feng Y, Yang Y, Han Q, et al. (2020). Transplantation of ACE2- Mesenchymal Stem Cells Improves the Outcome of Patients with COVID-19 Pneumonia. Aging Dis, 11:216-228. 32257537 10.14336/AD.2020.0228 PMC7069465

[19] Liang B, Chen J, Li T, Wu H, Yang W, Li Y, et al. (2020). Clinical remission of a critically ill COVID-19 patient treated by human umbilical cord mesenchymal stem cells: A case report. Medicine (Baltimore), 99:e21429. 32756149 10.1097/MD.0000000000021429 PMC7402800

[20] ARDS Definition Task force Berlin, Ranieri VM, Rubenfeld GD, Thompson BT, Ferguson ND, Caldwell E, Fan E, et al. (2012). Acute Respiratory Distress Syndrome. The Berlin Definition JAMA, 307:2523-2533. 10.1001/jama.2012.5669 22797452

[21] Hansell DM, Bankier AA, MacMahon H, McLoud TC, Müller NL, Remy J (2008). Fleischner Society: Glossary of Terms for Thoracic Imaging. Radiology. 246: 697-722. 18195376 10.1148/radiol.2462070712

[22] Barkama R, Mayo M, Paz A, Solopov A, Mann T, Vadasz Z, et al. (2020). Placenta-Derived Cell Therapy to Treat Patients with Respiratory Failure Due to Coronavirus Disease 2019. Crit Care Expl, 2:e0207. 10.1097/CCE.0000000000000207 PMC7498138 32984833

[23] Perlee D, Van Vught LA, Scicluna BP, Maag A, Lutter R, Kemper EM et al. (2018). Introvenous infusion of human adipose mesenchymal stem cells modifies the host response to lipopolysaccharide in humans: A randomized sinlge-blind, parallel group, placebo controlled trial. Stem Cells, 36: 1778-1788. 30063804 10.1002/stem.2891

[24] Laffey JG, Matthay MA (2017). Fifty Years of Research in ARDS. Cell-based Therapy for Acute Respiratory Distress Syndrome. Biology and Potential Therapeutic Value. Am J Respir Crit Care Med, 196:266-273. 28306336 10.1164/rccm.201701-0107CP PMC5549868

