# Supplementary material for: Mesenchymal Stem Cells for the Compassionate Treatment of Severe Acute Respiratory Distress Syndrome Due to COVID 19
Source: Aging Dis. 2021 Apr 1;12(2):360–70. doi: 10.14336/AD.2020.1218 (PMC7990366; doi:10.14336/AD.2020.1218)
Supplement: Supplementary file 1 [file AD-12-2-360-s.pdf]

SUPPLEMENTARY DATA

Mesenchymal Stem Cells for the Compassionate Treatment of Severe Acute Respiratory Distress Syndrome Due to COVID 19

Martin Iglesias[1,9,*], Patricia Butrón[1], Iván Torre-Villalvazo[2], Erik A. Torre-Anaya[2], Juan Sierra-Madero[3], José J. Rodriguez-Andoney[4], Armando R. Tovar-Palacio[2], Alejandro Zentella-Dehesa[5], Guillermo Domínguez-Cherit[4,9], Tatiana S. Rodriguez-Reyna[6], Julio Granados-Arriola[7], Verónica Espisosa-Cruz[8], Fernando P. Téllez-Pallares[10], Alexia Lozada-Estrada[10], Carol A. Zepeda Carrillo[10], Aldo J. Vázquez-Mézquita[11], Hector F. Nario-Chaidez[12]

© 2020. Iglesias M et al. Published online at http://www.aginganddisease.org/EN/10.14336/AD.2020.1218

SUPPLEMENTARY DATA

CLINICAL CASES

Patient 1

A 34-year-old man without a significant medical history developed a cough, general malaise, runny nose, and fever (37.5°C) on 30 April 2020. He visited several physicians who administered intramuscular dexamethasone at 8 mg every 24 hours on three occasions with partial improvement. On 3 May 2020, the patient developed weakness and fatigue associated with fever and dyspnea. On 5 May 2020, he additionally developed respiratory distress. For this reason, the patient was sent to our institution and hospitalized on 6 May 2020. Computed tomography (CT) showed COVID-19–compatible pneumonia with severe condition (pneumonia affecting >50% of the lung fields). He was initially treated with tocilizumab, azithromycin, amoxicillin, and enoxaparin without improvement. He was intubated in the intensive care unit (ICU) on 9 May 2020. The patient's partial pressure of arterial oxygen/fraction of inspired oxygen ratio (PaO_2/FiO_2) decreased to 76 mmHg with a body temperature of 37.6ºC, heart rate (HR) of 93 beats/min, and respiratory rate (RR) of 30 breaths/min. His baseline D-dimer concentration was 397 ng/mL and subsequently increased to 1373 ng/mL. His total lymphocyte count was 830/µL.

The patient underwent infusion of allogeneic human umbilical cord mesenchymal stem cells (ahUCMSCs) on 26 May 2020. At 24 hours after the ahUCMSC infusion, he exhibited an increase with a linear trend in the PaO_2/FiO_2 value, which continued to increase until reaching 179 mmHg on the ninth day post-infusion. Therefore, he was extubated, and an oxygen mask was placed. He received oxygen at 5 L/min, and his oxygen saturation (SpO_2) was 96%. His body temperature decreased after 24 hours and continued decreasing until it normalized on the third day. His HR also decreased and was maintained at an average of 72 beats/min. The patient's D-dimer concentration increased during the first 24 hours after the infusion of ahUCMSC, until reaching 2270 ng/mL and remained elevated because he developed metabolic ileus on 30 May 2020, at which time it reached 3272 ng/mL. Consequently, his RR also increased during those days, but it normalized at the end of the seventh day. His C-reactive protein concentration (5.85 mg/L) and procalcitonin concentration (1.18 ng/mL) were increased because of metabolic ileus as well as bacterial pneumonia associated with *Acinetobacter ursingii*. This infection was treated with meropenem. During the patient's clinical course, the total lymphocyte count increased from 830 to 2020/µL. Other medications given after the ahUCMSC infusion were metronidazole and vancomycin. The patient was discharged from the ICU on 5 June 2020 and continued his treatment in the hospitalization area. He was discharged from the hospital 2 weeks later. Table 1-4, Figure 1-4, and Supplementary Fig.1.

Patient 2

A 58-year-old woman developed general malaise, myalgia, arthralgia, odynophagia, and a fever (38.0°C) on 14 May 2020. She was initially treated with moxifloxacin and ipratropium/albuterol. On 17 May 2020, her symptoms progressed to perioral cyanosis and dyspnea on minimal exertion, necessitating oxygen therapy; however, her SpO_2 continued decreasing to 60%. She was sent to our institution and hospitalized on 19 May 2020. Simple chest CT showed COVID-19–compatible pneumonia and severe condition. Her PaO_2/FiO_2 decreased to 102 mmHg, and she was therefore intubated in the ICU. Despite general symptomatic treatment and administration of ceftriaxone, azithromycin, meropenem, vancomycin, trimethoprim/sulfamethoxazole, and enoxaparin, the patient's condition worsened (PaO_2/FiO2 of 84 mmHg, body temperature of 36.5ºC, HR of 56 beats/min, and RR of 24 breaths/min). Her baseline D-dimer concentration was 692 ng/mL and increased to 1765 ng/mL during her stay in the ICU, with a total lymphocyte count of 540/µL.

The patient underwent ahUCMSC infusion on 26 May 2020. She subsequently showed a progressive linear increase in her PaO_2/FiO_2 from the first 24 hours after infusion until the ninth day, at which time her PaO_2/FiO_2 reached 172 mmHg. Like Patient 1, she was extubated at this time point. Bacterial pneumonia was diagnosed at 48 hours post-infusion, and the patient developed a fever of 38.0ºC. She was treated with piperacillin/tazobactam because no specific bacteria were detected. The infectious process was controlled during the next 3 days. At 12 days post-infusion, the patient's vital signs were normal and her PaO_2/FiO_2 was 199 mmHg. Her D-dimer concentration transiently increased to 3159 ng/mL at 24 hours after the infusion; it then progressively decreased until it reached 1317 ng/mL at the end of

© 2020. Iglesias M et al. Published online at http://www.aginganddisease.org/EN/10.14336/AD.2020.1218

follow-up. Her lymphocyte count increased from 540 to 1531/μL. The patient was discharged from the ICU 12 days after the infusion and discharged from the hospital 4 days later (Table 1-4, Figure 1-4, Supplementary Fig. 1).

Patient 3

A 67-year-old man presented to the emergency room on 19 May 2020 with a fever (37.1ºC), respiratory distress, blood pressure of 102/60, HR of 95 beats/min, RR of 30 breaths/min, and SpO_2 of 85%. A CT scan showed bilateral pneumonia, and he was therefore hospitalized in our institution. His treatment involved supportive care, supplemental oxygen, and enoxaparin. On 20 May 2020, he developed refractory hypoxemia with a PaO_2/FiO_2 of 89 mmHg, HR of 90 beats/min, RR of 28 breaths/min, and body temperature of 37ºC. At this moment the simple chest CT showed COVID-19–compatible pneumonia and severe condition. Tracheal intubation and ICU admission were indicated; however, the patient refused this treatment. Because of his hypoxemia, he was administered one dose of methylprednisolone (1 mg/kg) and placed in the prone position on 24 May 2020. Despite this treatment, his PaO_2/FiO_2 decreased to 62 mmHg. His D-dimer concentration on admission was 2390 ng/mL and subsequently increased to 3809 ng/mL, with a total lymphocyte count of 369/μL.

The patient underwent ahUCMSC infusion as compassionate treatment on 27 May 2020. At 24 hours post-infusion, his PaO_2/FiO_2 increased to 84 mmHg but his poor medical condition persisted. Although the patient agreed to undergo intubation at this time, no beds were available in the ICU. His PaO_2/FiO_2 showed linear improvement until reaching 188 mmHg on 2 June 2020; therefore, his oxygen administration was decreased to 10 L/min and he was placed in the supine position. Only three doses of methylprednisolone were administered (29 May, 2 June, and 3 June 2020) because of hyperglycemia and improvement in his PaO_2/FiO_2. The patient's HR and RR normalized at 24 hours and his body temperature normalized at 48 hours. His D-dimer concentration increased in the first 24 hours to 4614 ng/mL, but it then decreased in the next 24 hours until it reached 1564 ng/mL at the end of follow-up. On 6 June 2020, the patient developed abdominal constipation that resolved with medical treatment. His medical status improved, and he was discharged on 16 June 2020 (Table 1-4, Figure 1-4, Supplementary Fig. 1).

Patient 4

A 62-year-old man developed fever, shortness of breath, a dry cough, and runny nose on 18 May 2020. He was treated medically at another institution but could not recall what type of treatment was given. On 27 May 2020, he presented to the emergency room of our hospital with a fever (38.0ºC), HR of 100 beats/min, RR of 28 breaths/min, PaO_2/FiO_2 of 101 mmHg, and troponin concentration of 103 pg/mL with negative precordial T waves. Simple chest CT showed COVID-19–compatible pneumonia and severe condition. He was admitted to the ICU, where he underwent tracheal intubation and placement in the prone position. Tazobactam, vancomycin, and enoxaparin were given. On 29 May 2020, his PaO_2/FiO_2 decreased to 86 mmHg and his D-dimer concentration was 1117 ng/mL, ferritin concentration was 5871 μg/L, total lymphocyte count was 943/μL, C-reactive protein concentration was 31.2 mg/L, and procalcitonin concentration was 2.15 ng/mL.

The patient underwent ahUCMSC infusion on 29 May 2020. The body temperature decreased during the first 24 hours post-infusion; however, it subsequently increased until it reached 38.5ºC and was maintained at this level for 15 days. His HR and RR increased to 106 beats/min and 28 breaths/min, respectively. His PaO_2/FiO_2 began to increase 24 hours post-infusion until reaching 158 mmHg on the fifth day post-infusion. At this time, the patient's PaO_2/FiO_2 began to decrease, reaching 123 mmHg on the ninth day post-infusion; however, the PaO_2/FiO_2 was still better than that in the pre-infusion stage. The D-dimer concentration increased to 4712 ng/mL after the first 24 hours post-treatment and then progressively decreased to 991 ng/mL until 8 June 2020. His lymphocyte counts progressively increased to 811/μL until 10 June 2020. At 48 hours post-infusion, the patient exhibited liver failure with an increased bilirubin concentration of 3.86 mg/dL, alanine aminotransferase concentration of 85.4 IU/L, and aspartate aminotransferase concentration of 130 IU/L. His C-reactive protein concentration was 32.75 mg/L and his procalcitonin concentration was 2.21 ng/mL at 48 hours post-infusion. On 6 June 2020, radiographs revealed progression of his pneumonia; therefore, he was treated with meropenem and vancomycin. On 11 June 2020, the patient's hemoglobin concentration decreased to 7.2 g/dL; his anticoagulation treatment was therefore suspended, and he underwent transfusion of 2 units of red blood cells. On 12 June 2020, he developed arterial thrombosis of the left lower limb and hemodynamic deterioration, and his D-dimer concentration reached 7268 ng/mL. He subsequently died. *Enterobacter cloacae* was cultured from an endotracheal aspiration specimen (Table 1-4, Figure 1-4, Supplementary Fig. 1).

© 2020. Iglesias M et al. Published online at http://www.aginganddisease.org/EN/10.14336/AD.2020.1218

SUPPLEMENTARY DATA

Patient 5

A 45-year-old man developed a fever (38.5ºC) on 25 May 2020. He was treated with ceftriaxone and acetaminophen for 7 days. On 31 June 2020, his fever was still present and a polymerase chain reaction test for SARS-CoV-2 was positive. He was treated at home with oxygen, four doses of methylprednisolone (500 mg), salbutamol every 12 hours, furosemide (40 mg) every 8 hours, and acetaminophen (500 mg) every 8 hours. On 5 June 2020, he developed dyspnea and cyanosis and presented to the emergency room of our institute. He was admitted to the ICU on 7 June 2020 with a fever of 38.0ºC, HR of 120 beats/min, RR of 50 breaths/min, and PaO_2/FiO_2 of 57 mmHg. Treatment with piperacillin/tazobactam and enoxaparin was started. His clinical course was poor despite the intensive care therapy. His initial D-dimer concentration was 1549 ng/mL, total lymphocyte count was 652/µL, and C-reactive protein concentration was 11.99 mg/L. Because the patient did not respond satisfactorily to medical treatment administered for 48 hours in the ICU, ahUCMSCs were infused on 9 June 2020.

After the infusion, the patient's fever remained at 38.0ºC and progressively increased until reaching 39.7ºC on the fourth day post-infusion; it thereafter remained above 38.7ºC during his entire stay in the ICU. His HR remained at 100 beats/min and RR decreased to 28 breaths/min. His PaO_2/FiO_2 increased in the first 24 hours to 150 mmHg; it was then 134 mmHg on 15 June 2020 and 138 mmHg on 17 June 2020. From this day on, the patient's PaO_2/FiO_2 progressively decreased until reaching 53 mmHg on 22 June 2020, the last day of his stay in the ICU. His D-dimer concentration rose in the first 24 hours post-infusion to 2738 ng/mL and then began to decrease until reaching 2038 ng/mL on 20 June 2020. His total lymphocyte count did not show great changes, amounting to 775/µL on 21 June 2020. His C-reactive protein concentration increased to 36.54 mg/L on the third day post-infusion and began to decrease until reaching 2.11 mg/L on 21 June 2020. His procalcitonin concentration progressively increased until reaching 19.31 ng/mL on 19 June 2020. Since his admission to the hospital, the patient's serum creatinine concentration progressively increased from 2.51 to 3.71, 5.02, and 7.14 mg/dL, at which time hemodialysis was performed; however, the treatment was incomplete because of hemodynamic alterations. On 20 June 2020 (11 days post-infusion), the patient developed bleeding from the nose and bladder, and his anticoagulation therapy was suspended. He died 13 days after the infusion (22 June 2020) (Table 1-4, Figure 1-4, Supplementary Fig. 1).

Supplementary Table 1. Cell Surface Markers of ahUCMSC

Cell Marker	Percentage %
Viability	99.95
CD34+	00.03
CD44+	99.29
CD45+	00.03
HLA-DR	00.10
CD73+	99.98
CD90+	99.96
CD105+	99.99

© 2020. Iglesias M et al. Published online at http://www.aginganddisease.org/EN/10.14336/AD.2020.1218

SUPPLEMENTARY DATA

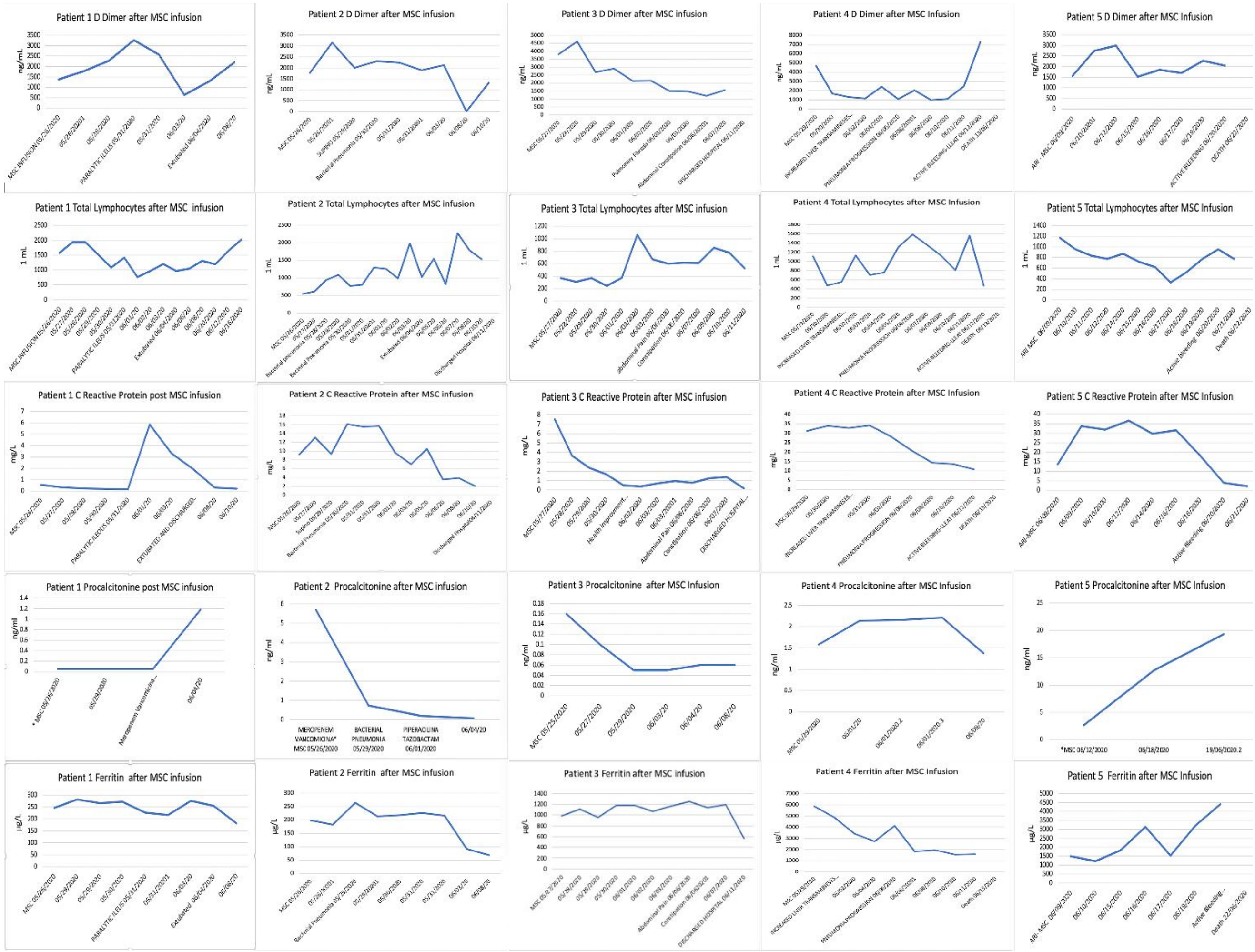

Supplementary Figure 1. Biochemical Changes.

© 2020. Iglesias M et al. Published online at http://www.aginganddisease.org/EN/10.14336/AD.2020.1218
